# Modeling of Control Efforts against *Rhipicephalus sanguineus*, the Vector of Rocky Mountain Spotted Fever in Sonora Mexico

**DOI:** 10.3390/insects13030263

**Published:** 2022-03-07

**Authors:** Gerardo Alvarez-Hernandez, Alejandro Villegas Trejo, Vardayani Ratti, Michael Teglas, Dorothy I. Wallace

**Affiliations:** 1 Departamento de Medicina y Ciencias de la Salud, Universidad de Sonora, Hermosillo 83067, Mexico; galvarezh63@gmail.com; 2 Consultoría para la Evaluación e Investigación en Salud Pública (CEISP), Cuernavaca 62440, Mexico; alex_villegas_trejo@hotmail.com; 3 Department of Mathematics, California State University, Chico, CA 95929, USA; vratti@csuchico.edu; 4 Department of Agriculture, Veterinary and Rangeland Sciences, University of Nevada, Reno, NV 89557, USA; mteglas@unr.edu; 5 Department of Mathematics, Dartmouth College, Hanover, NH 03755, USA

**Keywords:** Rocky Mountain spotted fever, *Rhipicephalus sanguineus*, *Rickettsia rickettsi*, tick-borne disease, tick control, insecticidal wall treatment, dog collars

## Abstract

**Simple Summary:**

Rocky Mountain spotted fever (RMSF) is among the most fatal of all bacterial diseases in the Americas. Humans become ill through the bite of ticks infected with the bacterium *Rickettsia rickettsii*. Several biological, environmental, and social determinants play a role in the occurrence of the disease, which has extended its presence throughout the region. To prevent this medical threat, innovative interventions has been implemented in some communities, although they still do not have a widespread application and have not been used in combination. In this study, we examined through mathematical models the potential benefit of combining insecticidal dog collars and long-lasting wall treatments to reduce the burden of ticks in a socially vulnerable Mexican community with a high burden of cases and deaths due to RMSF. Overall, we found that if enough coverage is given for either treatment, the other one can be omitted. Both interventions have the potential to lessen the burden of ticks and may help to lower the risk to be ill from RMSF in communities such as of our study. We recommend further research including some other factors (i.e., political, budgetary, socioeconomics) linked to the disease.

**Abstract:**

Rocky Mountain spotted fever (RMSF) is a significant health problem in Sonora, Mexico. The tick vector, *Rhipicephalus sanguineus*, feeds almost exclusively on domestic dogs that, in this region, also serve as the reservoir for the tick-borne pathogen, *Rickettsia rickettsii*. A process-based mathematical model of the life cycle of *R. sanguineus* was developed to predict combinations of insecticidal dog collars and long-lasting insecticidal wall treatments resulting in suppression of indoor tick populations. Because of a high burden of RMSF in a rural community near the Sonora state capital of Hermosillo, a test area was treated with a combination of insecticidal dog collars and long-lasting insecticidal wall treatments from March 2018 to April 2019, with subsequent reduction in RMSF cases and deaths. An estimated 80% of the dogs in the area had collars applied and 15% of the houses were treated. Data on tick abundance on walls and dogs, collected during this intervention, were used to parameterize the model. Model results show a variety of treatment combinations likely to be as successful as the one carried out in the test community.

## 1. Introduction

Rocky Mountain spotted fever (RMSF) is a deadly disease. The reported fatality rate can be as high as 30–80% in some areas if specific treatment is not initiated in time [1,2,3,4]. Early symptoms of the disease include fever, headache, rash, and malaise. Clinical complications can produce a myriad of adverse outcomes such as hemorrhage, purpura, necrosis, hepatic failure, acute kidney injury, meningismus, and cardiopulmonary involvement, among others [5,6,7,8,9,10]. Critically ill patients may suffer organ failure and those who recover may develop long-term consequences [1,11,12]. Although isolated cases and familial clusters can appear throughout the Americas [1], localized Rocky Mountain spotted fever outbreaks can occur, as in some communities in northern Mexico [5,13,14,15]. Cases have been reported in the region since 1940 [1]. A high incidence of cases is also reported in Arizona tribal lands [16]. The disease is caused by the bacteria *Rickettsia rickettsii*, transmitted in Mexico, through the bite of its main tick vector, *Rhipicephalus sanguineus* [17].

Like other hard ticks, *Rh. sanguineus* requires three blood meals to complete its life cycle of egg, larva, nymph and adult. This species feeds almost exclusively on domestic dogs for all three of these blood meals, with dogs serving as a disease reservoir for RMSF. Although highly adapted to indoor living, these ticks also survive outdoors in compatible climates or with protective refuges. Indoors it can hide in cracks and crevices during periods when it is not actively seeking a host [18]. Maturation times and death rates for the life stages are variously affected by temperature and humidity, which accounts for most of the seasonal variation in abundance [18,19,20,21].

In the same community as the one studied here, a previous investigation in 2016 used dog collars and peri-domestic acaricide to reduce tick abundance, with the result that no new human cases were reported during the 18-mo. period of intervention [22,23]. In those studies, interventions included the extensive use of acaricide dog collars in Community A, while in Community B acaricide treatments were used extensively without the use of dog collars. This study focuses on tick populations observed on both walls and dogs of Community A, in which dog collars were combined with three types of acaricidal wall treatments, and with visual inspections of houses and dogs used to track changes in tick abundance.

The model developed in this study is based on prior models of *Ixodes scapularis* and revised to include temperature and humidity responses, and other development parameters particular to *R. sanguineus*, and include the appropriate host structure [24,25,26]. *Ixodes sp.* can undergo diapause for a variety of reasons, both external and “behavioral”. Other studies indicate that most seasonal patterns of *R. sanguineus* can be accounted for by temperature extremes, with a lack of development below 10 ${}^{{}^{\circ}}$C in unheated dog kennels, and observed winter diapause [19,27,28]. The ability to undergo diapause has been suggested as one reason for the latitudinal expansion of this species [29]. In addition to temperature driven diapause, *R. sanguineus* exhibits diapause under long daylight (16:8 hr) conditions [30]. The study region in Sonora, Mexico, rarely drops to 10 ${}^{{}^{\circ}}$C in temperature and maximal day length does not quite reach 16 h. In addition, the ticks in the study area are in human houses, so temperature is further modulated. For these reasons, the model developed in this study does not take diapause into account.

The purpose of this analysis is two-fold. Firstly, we compare the effectiveness of the three different insecticidal wall treatments in the presence of the dog collar intervention. Secondly, we use the model developed to identify other combinations of dog collaring and insecticidal wall treatment likely to produce equal or better results at reducing tick abundance.

## 2. Materials and Methods

The methods used comprise both an epidemiological intervention and a mathematical simulation. The epidemiological intervention was carried out in a small rural community; in 2016 with the placement of collars with insecticide and residual spraying with deltamethrin and later in 2018, two different applications of insecticidal paint for walls, residual spraying with Propoxur and a control group. The mathematical simulation is built on a process-based model designed to predict results of different coverages of dog collar and wall treatment.

### 2.1. Epidemiological Intervention

The rural community chosen for participation in this study was considered to be at high risk for RMSF, and included 643 houses, with a substantial migrant worker population. Dogs were observed moving freely inside and outside of houses and were free-roaming throughout the community. Houses with dogs were determined by the presence of dogs inside or outside of the house and the number of animals present was recorded. Investigators observed ticks on the walls of domestic dwellings as well as outdoors in peri-domestic areas prior to and during the intervention. Anywhere from 2 to 35 dogs were sampled from each intervention category on each sampling trip.

Prior to the intervention, a census was taken of the dog population, counting approximately 1250 dogs in the intervention area. Of these, a thousand dogs were collared initially, representing approximately 80% of the dog population. Another 111 were collared during the first three months of intervention and dogs found to have lost their collar were re-collared. Some dogs were not collared or were re-collared because of the risk of being bitten, or because the owners were not at home. Lost collars were also replaced during May, August and October, but such a replacement was done only when it was observed that a dog did not have one (i.e., it was lost, it was removed by its owners). There was not a systematic replacement of collars lost on the entire population of those collared at the start of the study. An estimated 10–15% of dogs in inspected houses had collars replaced during the study. At the end of a year of study, 30% of the total collars placed (about 333) were observed to have been lost. Bayer (Bayer Animal Health, Leverkusen, Germany) Seresto tick collars were used in the study. These contain 4.5% flumethrin and 10% imidacloprid, and are estimated to control ticks for 7–9 months according to various studies [31,32]. Dogs in untreated houses were collared as well as those in treated houses, thus the term “control” refers, in this study, to houses in a treatment area where approximately 80% of dogs were collared but no wall treatment applied.

Three types of acaricide treatment were used: indoor residual spray (IRS-PPX) initially followed by propoxur wettable powder 1% (Codequim Proxur 1%), insecticidal paint on all walls to a height of 1 m. (WIP1m), and insecticidal paint on all walls to full height (WIP). Houses initially assigned to the “control” group received treatment when the tick infestation in a house was so high that it was decided to apply insecticidal treatment, and an alternate house was assigned to the control group.

*Indoor residual spray followed by propoxur wettable powder.* During 2016, residual spray treatments were applied in 140 homes, both indoors and outdoors, representing approximately 22% of the 643 houses in the study area [22]. Indoor residual acaricide containing 5% deltamethrin (Bayer K-Othrine WG250) was applied to the interior of homes, in the IRS intervention, by trained personnel of the Ministry of Health. These houses were continued in the current study by professional application of propoxur wettable powder (Codequim Proxur 1%) with the oversight of licensed pest applicators, and reapplied according to the product label.

*Insecticidal paint.* Safecolor (Codequim, RSCO-USP-39-2016) insecticidal wall paint was used in the WIP1m and WIP interventions. This product contains a slow-release formula of 1% Propoxur developed by Inesfly. The manufacturer claims that the insecticidal effect persists for 2 years on interior walls. Between March and April 2018, insecticidal paint was applied on all walls at a height of 1 m in one treatment group (WIP1m), or insecticidal paint on all walls to full height inside each house in another treatment group (WIP).

A sample of houses in each of the four treatment categories (Control, IRS-PPX, WIP1m, WIP) was surveyed nine times from March 2018 to April 2019 (3/26/18, 5/8/18, 5/25/18, 6/20/18, 7/18/18, 8/18/18, 10/30/18, 1/29/19, 4/30/19). Ticks found on walls were recorded by life stage in houses with and without dogs. In houses with dogs, the ticks on dogs were reported by life stage during each inspection in the study. Homes with dogs had an average of 2.36 dogs present per house. Tick inspection of houses in each treatment category was performed monthly but the timing of the inspections was based on the availability of resources and whether the inhabitants were at home at the time of inspection. Tick sampling inside houses was performed in a routine manner with a thorough visual inspection of 30 min per house. The number of ticks of each life stage was recorded per inspection and for each location. Sampling of ticks on dogs was performed in an opportunistic manner and was based on whether dogs were present and if the animals could be handled safely during the examination process. The ticks collected from each dog were identified by species and life stage, and the numbers recorded.

Adult ticks present in houses, in houses with dogs, and on dogs, were compared among treatments using data from inspections days 2–8 after treatments were started. Adults ticks are more visible and researchers found more of them, and so are a more reliable measure treatment comparison. The first inspection was considered baseline data and not reflective of the results of treatment, and so was omitted. It is not recorded whether tick infested houses had dogs or not, but one might expect that most ticks would be in houses with dogs. To account for this possibility, treatment comparisons were made on (1) adult ticks per house, (2) adult ticks per house with dogs, (3) adult ticks per dog. The four treatments (control, WIP1m, WIP, IRS-PPX) were compared using a one-way ANOVA test followed by Tukey’s HSD.

Approval was obtained from the Institutional Review Board of the Universidad de Sonora. All the procedures were performed after ethical approval as well as traditional authorization from the native local authorities.

### 2.2. Model Development, Parameterization, and Numerical Simulations

**Figure 1:**
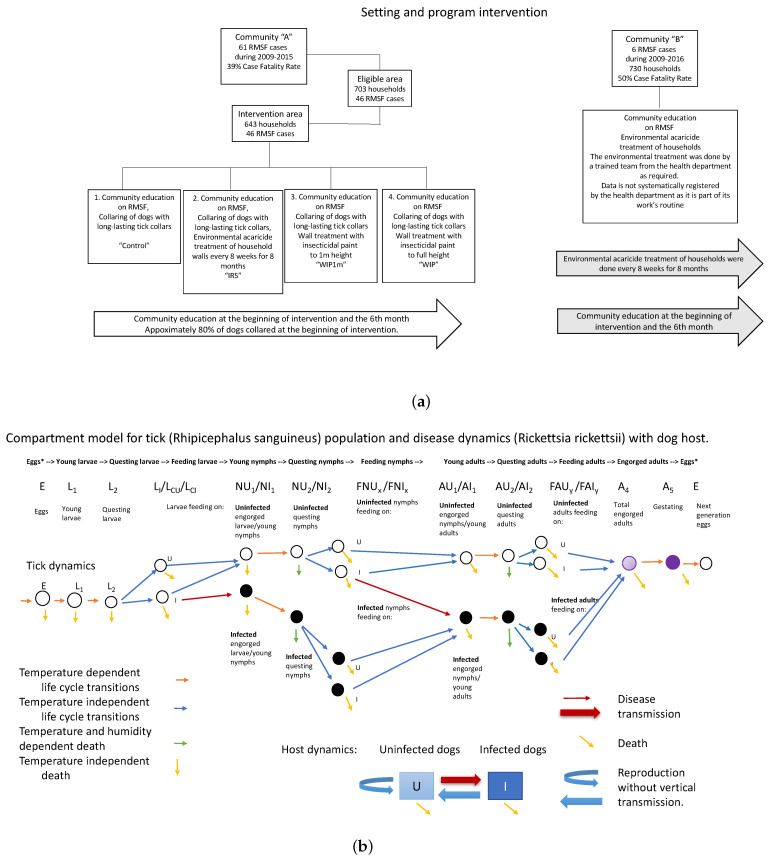
(**a**) A diagram of the overall study plan for communities A and B. (**b**) Compartment model for accompanying process-based model. Orange and green arrows indicate temperature or temperature and humidity dependent transitions. Black compartments are infective ticks.

The process based model used to investigate multiple intervention strategies is based on a prior model of the tick vector for Lyme Disease, *Ixodes scapularis* [24,25,26]. Dynamic compartments for maturing, questing, and feeding larvae, nymphs and adults were included for both infected and uninfected populations, as well as population dynamics of host species, represented as a system of ordinary differential equations. That model incorporated temperature dependent maturation rates, a temperature profile for Hanover, New Hampshire, and multiple types of host. The model was revised for *R. sanguineus* by replacing the temperature simulation and maturation dependencies, including a new humidity simulation and dependency for the death rate of questing nymphs, and revising the host populations to be only susceptible and infected domestic dogs. The compartment model thus modified is illustrated in Figure 1. All differential equations, supplementary equations, and parameters are in Appendix A.

A study by Koch and Tuck [20] measured development times and survival for maturing *R. sanguineus* larvae and nymphs, as well as death rates of unfed nymphs and adults, under varying conditions of temperature *T* and humidity *H*. It is clear from the data that humidity was not a large factor in maturation rates of fed larvae and nymphs, however, temperature dependence was considerable. For fed larvae and nymphs, reported values were averaged over humidity values for each temperature recorded.

**Figure 2:**
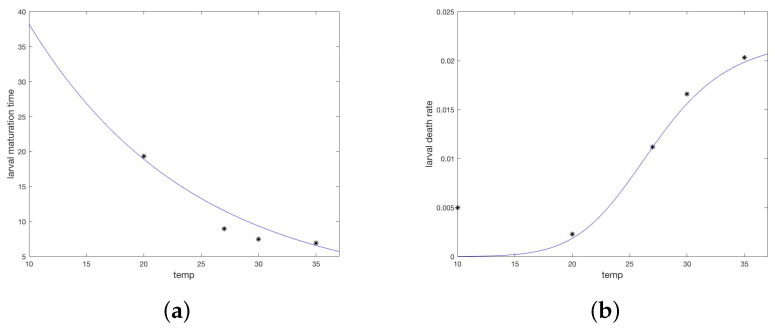

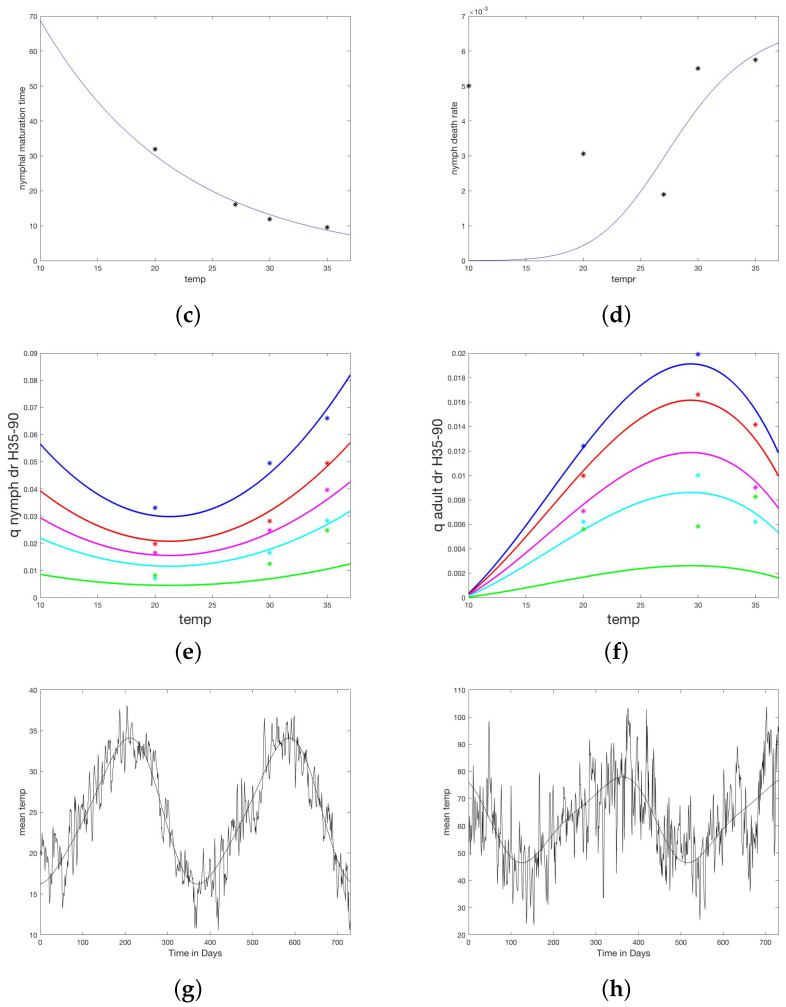
Temperature and humidity dependent responses of maturation and death times or rates fit to data from Koch and Tuck [20]. (**a**) Larvae maturation times as a function of temperature. (**b**) Larvae death rate as a function of temperature. (**c**) Nymph maturation times as a function of temperature. (**d**) Nymph death rate as a function of temperature. (**e**) Unfed (questing) nymph death rate as a function of both temperature and humidity. Each curve represents a humidity level from bottom (90) to top (35%). (**f**) Unfed (questing) adult death rate as a function of both temperature and humidity. Each curve represents a humidity level from bottom (90%) to top (35%). (**g**) Fourier approximation to temperature data for the study site. (**h**) Fourier approximation to humidity data for the study site.

Average molting time tm was plotted against temperature and a regression line fit, yielding average larval molting time $tm_{L}=-0.0704*temp+4.2471$ ($R^{2}=0.88$) and average nymphal molting time, $tm_{N}=-0.0826*T+5.0564$ ($R^{2}=0.97$). These values were interpreted as a half life for the respective molting populations, giving maturation rates for larvae and nymphs respectively as follows and illustrated in Figure 2a,b.

(1) $$m_{L}=log\left(2\right)*e^{\left(-(-0.0704T+4.2471)\right)}.$$

and

(2) $$m_{N}=log\left(2\right)*e^{\left(-(-0.0826T+5.0564)\right)}.$$

Average time to molt tm and the percent molted *p* were converted to a daily death rate, *d*, as d=ln(100/p)/tm. When regressed against temperature, death rates follow linear models (not illustrated).

(3) $$d_{L}=\left(0.0012T-0.0221\right),R^{2}=0.98.$$

(4) $$d_{N}=\left(0.0002T-0.0018\right),R^{2}=0.49.$$

For unfed (off-host and ready to quest for a host) nymphs and adults, the data from Koch and Tuck show a strong dependence on both temperature and humidity. For each temperature and humidity, the authors report percent survival over a series of days. The median survival, *S*, was chosen and death rate computed as ln(2)/S. The death rates for unfed nymphs, $d_{UN}$ and adults, $d_{U}A$, were modeled as functions of temperature *T* and humidity *H* using the nonlinear fit device offered by Matlab (fitnlm, [33]). This fit was tested on several families of functions until a reasonable fit was achieved with a function that did not drop below zero in the temperature domain of interest (10–90 ${}^{{}^{\circ}}$C). Death rates for questing larva were taken equal to those for questing nymphs. The resulting relationships are given below and shown in Figure 2c,d.

(5) $$d_{UN}\left(T,H\right)=\left(0.24783T^{2}-10.539T+147.18\right)/\left(H^{2}-82.833H+2852.5\right),$$

$$rmse=0.0032,R^{2}=0.974.$$

(6) $$d_{U}A=T\left(-0.0030434T^{2}\right)+0.15459T-1.2034)/\left(H^{2}-93.456*H+3139.6\right),$$

$$rmse=0.00139,R^{2}=0.925.$$

Daily weather measurements from ERA5, for the two years of the study and region of intervention in Sonora (28.85 lat., −111.50 long.), were downloaded [34]. Average daily temperature data was fit with a truncated Fourier series using Matlab software [33]. Daily average relative humidity was calculated from ERA5 data for temperature, wet bulb temperature, and station pressure according to standard methods [35], then fit with a truncated Fourier series using Matlab software [33]. The resulting formula are given in Table A1 and the fit to data are shown in Figure 2e,f.

Other population parameters were drawn as necessary from the study by Ioffe-Uspensky et al. (1997), with the exit rate of ticks from the feeding compartments based on stage-dependent feeding times, as well as average clutch size and exit rate of the pre-oviposition period, which was corroborated by a second study [18,36]. Egg hatching rates are known to be temperature dependent, however not enough data was found to give a response curve [37], so the rate was borrowed from a prior modeling study [24]. Median time of larval hardening was taken from Koch and Tuck, and interpreted as a half-life [20]. On-host carrying capacity for nymphs and adults was taken to be 50, which is intermediate between the highest observed values and more commonly observed abundances.

The daily birth rate of dogs was estimated from an average 10 pups per year per female dog $b_{H}=0.0135$. The death rate for healthy dogs was based on a 10 year lifespan, giving $d_{H}=0.0002739726$. The death rate for infected dogs $d_{HI}$ is not reported, nor is a “carrying capacity”, $K_{H}$. These two were adjusted so that $d_{HI}=0.005479$, based on an average life expectancy of 6 months for infected dogs (without treatment, which is not included in this model), and $K_{H}=2000$ dogs, giving an equilibrium population of 1271 dogs close to the 1250 dogs estimated to be in the study area. Dog carrying capacity and transmission rates for larvae $p_{L}$, $nymphsp_{N}$, and dogs $p_{UI}$, were adjusted to give disease prevalence values for both dogs and ticks in reported ranges [38,39,40,41]. Disease prevalence in dogs and ticks was not measured in the intervention. Except for carrying capacity, $K_{H}$, all units are in percent change per day.

Cd = 50;

Death due to insecticidal wall treatment was modeled by a term of the form

(7) $$-d_{WT}X=-jXHV_{\tau }.$$

For all populations, *X* of off-host ticks, excluding eggs and hardening larvae which are likely to be sequestered in crevices. $HV_{\tau }$ is the Heaviside function at time ${}_{\tau }$ which is set to begin after steady state is reached. All treatments used in the epidemiological study were long-lasting or repeated regularly, so the death rate given by *j* is assumed to persist for a full year. In addition, the treatments killed ticks fairly quickly. Therefore, the parameter *j* mostly accounts for coverage, which should not be taken as equal to percent of walls treated in the community. Ticks that are not on hosts are not necessarily on walls; they can also be found on floors, furniture, outdoors, etc., mostly in peridomestic areas. For this reason, *j* will always be taken as less than one and must be interpreted as the percent of off-host ticks that are exposed to treated surfaces for sufficient time.

On-host feeding ticks are effectively killed by the dog collars used, according to manufacturers.

This effect is applied by a similar term of the form

(8) $$-d_{DC}Y=-cYHV_{\tau }.$$

This term is applied to all populations *Y* of feeding ticks. As in the case of wall treatments, c≤1 represents the level of coverage and should be interpreted as the percent of on-host ticks exposed to sufficiently toxic dogs for sufficient time.

A table of parameters used in all simulations is in Appendix A.3.

### 2.3. Numerical Simulations

From the intervention data in Table 1 and Appendix B, researchers found more adults than nymphs on walls and dogs. This is biologically unrealistic, as there must be many more juveniles than adults in a population with comparable maturation times for each stage, as is the case here. Adults are larger and easier to find and identify than younger, smaller juvenile stages. In addition, researchers found more ticks on dogs than on walls, contradicting both the model and estimates in the literature [42]. Therefore adult populations were used as points of comparison for simulations and on-host counts were considered more reliable than on wall counts.

**Figure 3:**
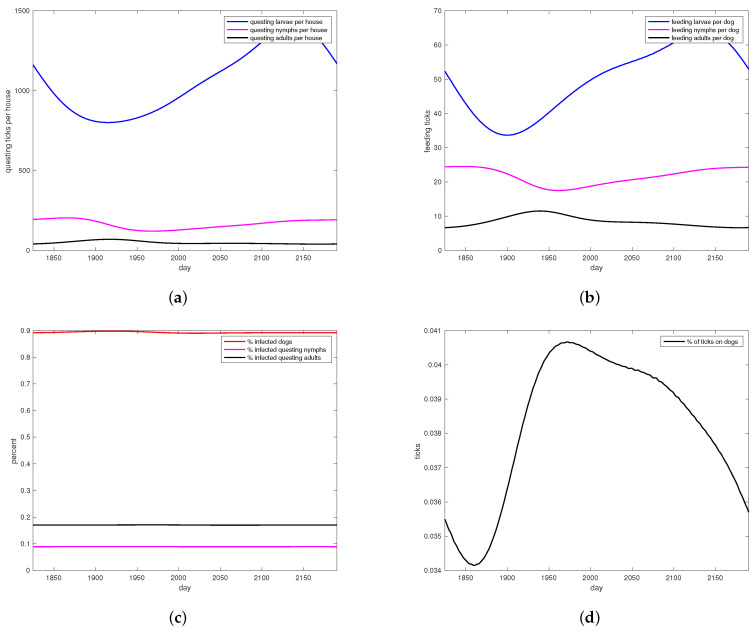
Simulations of tick abundance and RMSF prevalence for no treatment. (**a**) Questing larvae, nymphs and adults per house, at year 6 steady state. (**b**) Feeding larvae, nymphs and adults per dog, at year 6 steady state. (**c**) RMSF prevalence patterns with no treatment. Percent infectious nymphs, adults, and dog hosts are shown. (**d**) Ratio of feeding ticks to all ticks in all stages, shown at year 6 steady state.

**Figure 4:**
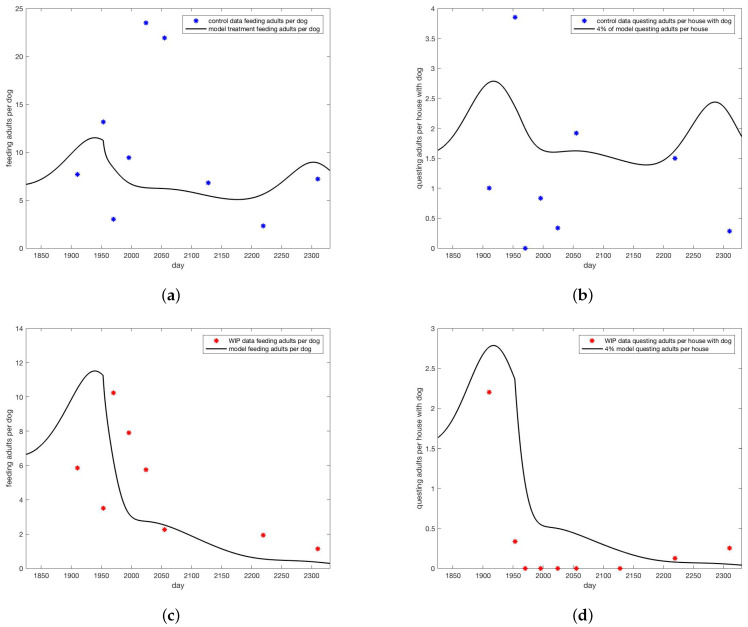
Model versus data for (**a**) adults on dogs for control and (**b**) on walls of houses with dogs for control. Data are blue dots, model trajectory is the black curve. For the data 80% coverage in collars was noted and no wall treatment. For the model, 8% coverage of dog collars (c=0.08) was used and no wall treatment (j=0). Model versus data for (**c**) adults on dogs for WIP full height insecticidal paint. Data are red dots, model trajectory is the black curve. (**d**) on walls of houses with dogs for WIP full height insecticidal paint. Data are red dots, model trajectory is the black curve. For the data 80% coverage in collars was noted and 15% wall treatment. For the model, 8% coverage of dog collars (c=0.08) was used and 3.75% wall treatment (j=0.0375). Note that for counts of ticks on walls, 4% of model value was shown for visual ease.

**Figure 5:**
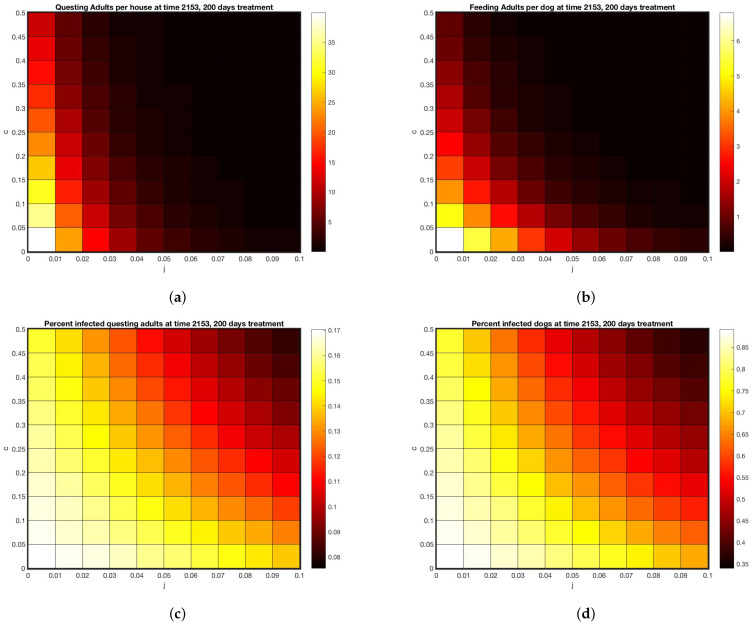
Model predictions under various treatment intensities or coverages for 200 days. (**a**) Abundance of questing adults per house (**b**) Feeding adults per dog (**c**) Percent of questing adults infected (**d**) percent of dogs infected.

All simulations were done using MatLab software [33]. Simulations with no treatment, shown in Figure 3 set c=j=0 with all other parameters as in Appendix A.1. Initial condition were set at 1 million eggs, 1248 uninfected dogs, one infected dog and run until steady state. The interventions included a “control” group which did have dog collars applied but no wall treatments. Simulations designed to track the data from the intervention were parameterized by first setting j=0 and selecting dog collar coverage *c* so that the model came within the range of the control data for on-host adults, shown in Figure 4a, giving c=0.05. For the WIP intervention, collar coverage *c* was kept the same and wall coverage *j* was increased until he model came within the range of the WIP data for on-host adults shown in Figure 4c, giving j=0.04. Heat maps shown in Figure 5 extended the range of *j* and *c*. In all cases where treatment was present in the model, it was applied at day 1953, corresponding to year 6 of the simulation after steady state is reached, day 128 of that year, corresponding to May 8, between the second and third survey visits when a decline in on-host ticks appears in the control group (see Appendix B).

## 3. Results

In total, 6944 *R. sanguineus* ticks were extracted and identified from 291 positive dogs (58.7%), out of a total of dogs 495 inspected on nine sampling dates. Likewise, during nine entomological samples, 406 *R. sanguineus* ticks were collected from 71 average homes reviewed in each sample. Of the total ticks captured in the positive houses, only 86 (21.1%) were found in the houses of both painting interventions, 104 (25.6%) corresponded to the residual spray intervention and 216 (51.7%) to the control house group. Summary data for tick counts in houses is given in Table 1. Data for tick counts on dogs is in Appendix B.

Over the nine data collection dates, the average number of adult ticks per dog and per house are given for each treatment along with standard deviations in Table 2.

The difference in means for days 2–9 was analyzed for mean adults per house, mean adults per house with dog, and mean adults per dog, using a one-sided ANOVA test followed by the Tukey HSD. All three results were significant in the ANOVA test at p<0.05. Significant comparisons are shown in Table 3.

More ticks were found on dogs during inspections than on walls of houses, as seen in Table 2. It is worth noting that larvae were seldom observed on walls of houses: only one time for the control houses and not at all for WIP interventions. More adults than larvae or nymphs were found in houses, as seen in Table 1 and on dogs, reported in Appendix B. For this reason, the mathematical model was adjusted to reflect counts of adult ticks found on dogs.

The presence of ticks on walls of houses is a better reflection of human risk of infection than ticks on dogs. As adult ticks were more readily observed than other stages, an ANOVA was used to determine treatment differences, followed by Tukey’s HSD for pairs, as seen in Table 3. Themean of adult ticks found on walls post-treatment in the “control” group was higher than in the WIP treatment group, and this difference was the only statistically significant difference found between the control and other treatments.

Figure 3 shows model performance at steady state, for a simulation with no intervention at all. This situation was not part of the field study but rather reflects the situation in the area before any intervention took place. Note that cohort stages are in reasonable ratios to each other (Figure 3a,b), and the percent of all ticks that are feeding ranges from 1% to over 4% (Figure 3d). Figure 3c shows simulation results for disease prevalence in nymphs, adults, and dogs at steady state.

In Figure 4, collaring and coverage parameters were adjusted to bring the model into the range of data for the control intervention of only collars and the WIP paint intervention (Figure 4). Much lower “treatment” rates were needed to bring the model into line with data than were reported for the real intervention. Reasons for this discrepancy are explored in the discussion. Treatment rates were based on adult ticks found on dogs. Note that model predictions of questing ticks greatly exceeded observed values.

Using the model parameters that fit treatment outcomes in Figure 3 and Figure 4 as a benchmark, the range of parameters was expanded for both collars and wall treatments in Figure 5.

## 4. Discussion

Rocky Mountain spotted fever is a medical threat and public health concern that needs to be addressed by means of innovative and comprehensive strategies, such as those carried out and modeled in this study. There is scientific evidence regarding the positive effect of applying insecticidal collars on dogs with a combination of imidacloprid/flumethrin to kill ticks, both in experimental and in-field investigations, but less is known about the simultaneous effect of combining collaring and insecticidal paint ([22,31,32]. Nor is it known what combinations of coverage levels lead to similar outcomes. In communities such as the one studied here, with many social disparities and a high burden of disease, mathematical modeling in conjunction with data collection is a tool that can identify a range of effective interventions. Of those identified, the medical team can choose the most sustainable or least expensive option.

The following questions remain to be addressed. How did the various test groups compare in terms of tick abundance? What issues arise with tick surveys and how much should we trust various types of data? How well did the model perform, both in relation to observations in the literature and with respect to data gathered in this study? What factors may influence discrepancies between model and data and how do these discrepancies inform future assumptions about treatment outcomes? Finally, what future work is warranted based on the outcome of this study?

### 4.1. Comparison of Test Groups

Similar percentages of dogs were collared in all treatment groups, so differences in tick abundance are most likely due to wall treatments. The wall paint at full height worked the best in terms of adult and nymphal ticks found on walls of houses, as seen in Table 3. Treatment groups had different performance in reducing the abundance of ticks on dogs, with indoor spray giving the lowest abundance. Ticks on walls are likely to be questing for a meal and represent the greatest risk to humans, whereas ticks on dogs are feeding and unlikely to bite those interacting with the dogs.

### 4.2. Challenges in Surveying Ticks Both on and off Host

Like most 3-host, Ixodid tick species, *R. sanguineus* spends the majority of its life in its environment under the direct influence of biotic (host availability, predators) and abiotic (temperature, precipitation) factors [38,39].The number of ticks infesting dogs can vary due to the density of dog hosts, by geography (higher mean infestations in tropical or semitropical regions versus temperate ones) and even seasonally [18]. For example, dogs living in the same geographic region had significantly higher numbers of *R. sanguineus* ticks infesting them during the dry season [43]. In the temperate regions of this tick’s range, host-seeking behavior (questing) is highest in the late spring, summer and early fall seasons.

Adults seem to be more readily seen when ticks in houses were counted, as seen in Figure 3a,b, even though it is biologically reasonable to expect smaller stages to be an order of magnitude greater in population than the larger ones. Larvae were seldom found on walls of houses. Given the larger size of adult ticks, it may not be surprising that this stage was observed most frequently in houses in this experiment. Ixodid ticks spend most of their life stages (94–97%) off their host [42,44]. Using this rule of thumb, one can estimate the number of ticks in a house from the number found on the dogs living in it and ticks per dog. Comparison of wall versus dog tick counts in the data do not come anywhere near this ratio. In fact, more ticks were often found per dog than per house with dog, as seen in Table 2.

However, the behavioral differences in the timing of detachment from the host (drop-off) between tick life stages may also account for our findings. Engorged *R. sanguineus* larvae exhibit a diurnal drop-off pattern (during the day when dogs are likely outdoors) while engorged nymphs typically undergo nocturnal detachment (at night when dogs are more likely to be indoors) [45,46]. Consequently, the engorged larvae molt and develop into questing nymphs outside while the engorged nymphs develop into questing adults inside the homes. In a study assessing RMSF risk factors in western Mexico, investigators counted the number of *R. sanguineus* ticks on dogs and inside houses and a similar pattern emerged. Nymphal stages accounted for 22.4% of the total number of ticks observed inside houses, the adult stages accounted for 75.9%, and larval stages constituted the smallest percentage identified (1.7% of total) [39].

### 4.3. Model Performance and Results

Model parameters for the treatment were derived from published measurements made independently of this study. Model results shown in Figure 3 are for populations with no treatment at all, which are not represented in the data. In Figure 3a, questing larvae populations are substantially greater than questing nymphs, which in turn are substantially greater than questing adults, as is expected. Questing ticks are present all year round, with only modest seasonal dynamics. Between 5 and 15 feeding adults per dog are produced by the model in Figure 3b, not too far from numbers reported in Appendix B. Disease prevalence rates for ticks shown in Figure 3c are within reported ranges for regions of high prevalence, however disease prevalence in dogs was a bit higher than the highest prevalence found in the literature [47]. Figure 3d shows predicted percentages of ticks that are feeding, which is in line with the observation that 3% to 6% of all ticks in the environment are on hosts [42,44]. In short, the model produces reasonable results compared to the literature, except for canine infection rates which are high.

As researchers found more adults than other stages, and more ticks on dogs than on walls, the count of adult ticks on dogs was used as the basis for model comparison with data. Collaring was represented in the model as a death rate for feeding ticks. In order to bring the model predictions for adults on dogs into the range of the data for the control group, shown in Figure 4a, it was necessary to make this rate 5% per day, substantially less than the reported 80% coverage for the intervention. Wall treatments were represented in the model as a death rate for all off-host ticks. To bring model predictions for adults on dogs into the range of the data for the WIP group, shown in Figure 4c, an off-host death rate of 4% per day was used. This rate is less than the estimated 22% coverage reported by researchers, but not as extreme a difference as the adjustment required for the on-host death rate.

Using these as a calibration, Figure 5 displays a range of outcomes 200 days after start of treatments, for varying tick death rates due to these two types of treatment. Treatment rates matched to feeding adult data are at (c=0.05, j=0.04). The model predicts between 5 and 10 questing adults per house for that combined treatment (off host death of 5% per day, on-host death of 4% per day). Using this benchmark, Figure 5a,b give a range of possible treatments yielding the same outcome, shown by the color scale. As coverage increases, the model predicts close to zero ticks at 50% on-host death and 10% off host death. The model predicts that if enough coverage is given for either treatment, the other one can be omitted.

However, even if coverage is raised to the highest levels shown in Figure 5, with 50% on host and 10% off host death rates, Figure 5c,d shows that disease prevalence persists in both the vector and the host, albeit at lower rates. The implication of this finding is that, even if visible ticks are very few, a bite from one of them has a chance of transmitting disease and should be taken seriously.

### 4.4. Factors Contributing to Discrepancies between Model and Data

The estimate given of 80% of dogs equipped with collars is probably high, for four reasons. First, we know that there was a large number of free-roaming dogs in the community where this experiment was performed, leading to underestimation of the rate of un-collared dogs. Entry and exit of dogs is not taken into account in the model, which assumes a closed system. Second, most dogs collared were not part of the subsequent tick surveys, so the majority of lost collars were probably not noticed or replaced, leading to decreased coverage over time. The model did not take decreased coverage into account, although that could be possible in future studies that track rates of collar loss. Third, a high rate of puppies was observed because sterilizing adult dogs was not part of the intervention, so it is possible that an unknown number of new dogs were un-collared. A high birth rate for dogs was included in the model, but it is not known how well it represents the dogs in the study community. Fourth, an unknown number of collars may have been removed by owners.

The model assumes perfect mixing of dogs and houses, which is not the case and can be seen in the data set, which separates houses with dogs from houses without dogs. The model would therefore be expected to predict better results from any treatment, which is why we compare possible treatment in Figure 5 to the parameters estimated from the data.

The discrepancy between the off-host death rate (5%) needed to match measurements of feeding adults in the WIP treatment and the reported coverage of 15% is easier to understand. Not all off-dog ticks are on walls, but could be on the floor, the yard, or under the furniture, all untreated peri-domestic areas. *R. sanguineus* has many ecological strategies even in absence of domestic hosts. The model suggests that two thirds of off-host ticks are in areas not reached by wall treatments. Given the low effective death rate of 4% with 15% wall coverage, Figure 5 should be interpreted with consideration of the difficulty of achieving death effective death rates of even 30%.

Although Rickettsia can be treated in dogs, the model did not include a recovery rate for dogs. This decision leads to high disease prevalence rates for dogs in the model, slightly higher than the very highest rate observed. The previously high prevalence of human RMSF in this community suggests that the prior disease prevalence in dogs could have been quite high. Data collected as part of the intervention did not include prevalence measures that could be used as a point of comparison. However, Figure 1a indicates that community education was a universal part of the intervention. Although the community was economically disadvantaged, it is possible that owners found a way to treat their dogs.

All of these discrepancies highlight the need to have better ways to estimate tick populations and better measures of dog and human behavior that can be incorporated into modeling efforts. In particular, researchers need to have control over free roaming dogs in communities targeted for RMSF interventions.

### 4.5. Future Work

The burden of morbidity and mortality due to RMSF remains extremely high compared with that of other regions, despite several interventions and years passed since its reemergence in early 2000. There are challenges to having success with this kind of intervention when the target population lives with deep social inequality, as in this study. A myriad of factors can explain such situation, i.e., (1) methodological flaws in the community control of ticks and hosts; (2) the lack of systematic evaluation of the medical and public health interventions carried out so far; (3) the scarcity of research done at the regional level to gain a better understanding of the vector and its hosts; (c) variables of social matter that should be addressed as well, for instance the political willingness and budget assigned to preventive interventions.

More accurate tick counts on walls could be achieved by full extermination of a recently occupied dwelling. An experiment of this sort could be carried out in infested dog kennels to get a better idea of the errors inherent in these measurements. Similarly, a study that identifies the percent of off-host ticks found on walls would lead to a more accurate estimate of coverage (in the sense of percent of walls treated) needed to produce the desired result. Such measures would allow a recalibration of the model to produce more accurate and easily interpreted results.

Further interventions, based on the findings here, can be informed by measures of the effects of interventions such as community education, veterinary care, and birth control. Models incorporating these can be used to estimate the expense and difficulty of various combinations of treatment, in addition to likely outcomes.

## 5. Conclusions

During the study period, no new cases of RMSF occurred in the intervened community. Both the data and the model show that, if one can decrease the tick burden, then an intervention can positively impact on health indicators. Both the data and model show that an intervention addressing all the sites and objects where ticks used to hide would warrant complete success. So, an integral approach may include collaring of dogs, painting of walls, spraying effective and safe pesticides, community education and neutering of dogs. Having a range of options that are likely to produce the same result, as the model in this case gives, is a useful tool for addressing these challenges to produce a sustainable solution.

This study demonstrates the need to translate and systematically implement scientific efforts to the public health arena. Although crucial in scenarios like this one, there remains a profound gap between science, integrating field trials and models, and effective public interventions against tick-borne disease.

## Figures and Tables

**Table 1:** *Rhipicephalus sanguineus* ticks collected from walls during home inspections by treatment (pre and post intervention) for the study period, 2018–2019; n= number of houses with dogs present during inspection.

Treatment	# Houses Inspected	# Houses with Tick Infestations	% Houses Inspected with Dogs Present (n)	*R. sanguineus* Larvae	*R. sanguineus* Nymphs	*R. sanguineus* Adults	*R. sanguineus* Total
**Pre Intervention**							
**Day 1**							
Control	19	2	80 (n = 12)	0	1	19	20
WIP1m	15	0	47 (n = 7)	0	0	0	0
WIP	15	4	80 (n = 12)	0	2	33	35
IRS-PPX	12	0	100 (n = 12)	0	0	0	0
Total	61	6		0	3	52	55
**Post-Intervention**							
**Days 2–9**							
Control	60	15	75 (n = 45)	45	28	119	192
WIP1m	53	9	58 (n = 31)	9	21	12	42
WIP	53	6	92 (n = 49)	0	6	5	11
IRS-PPX	55	11	75 (n = 41)	45	17	42	104
Total	221	41		99	72	178	349

**Table 2:** Average adults found per dog, per house, and per house with dogs, days 1–9.

Treatment Group	Mean Adults Per Dog (SDpop)	Mean Adults Per House (SDpop)	Mean Adults Per House With Dog (SDpop)
Control	10.56 (7.17)	1.50 (1.36)	1.89 (1.54)
WIP1m	21.31 (14.91)	0.18 (0.29)	0.278 (0.416)
WIP	5.71 (3.74)	0.32 (0.67)	0.384 (0.806)
IRS-PPX	3.16 (3.52)	0.65 (1.20)	0.708 (1.195)

**Table 3:** Statistically significant comparisons (Tukey HSD).

Comparison	WIP1m	WIP	IRS-PPX
Control	Adults Per House With Dog: Control > WIP1m (p=0.03)	Adults Per House: Control > WIP (p=0.04) Adults Per House With Dog: Control > WIP (p=0.014)	
WIP1m		Adults Per dog: WIP1m > WIP (p=0.03)	Adults Per dog: WIP1m > IRS-PPX (p=0.009)

## Data Availability

As the study was not granted with Mexican public funds, the databases are not required to be published in any public repository.

## Appendix A. Model Details

### Appendix A.1. Differential Equations

Eggs, *E*

(A1) $$\frac{dE}{dt}=bA_{5}-m_{e}E-d_{e}E,$$

Young, hardening larvae, $L_{1}$

(A2) $$\frac{dL_{1}}{dt}=m_{e}E-d_{UL}L_{1}-m_{1}L_{1},$$

Questing larvae, $L_{2}$

(A3) $$\frac{dL_{2}}{dt}=m_{1}L_{1}-d_{UL}L_{2}-m_{2}L_{2}-d_{WT}L_{2},$$

Larvae feeding on uninfected host, $L_{U}$

(A4) $$\frac{dL_{U}}{dt}=m_{2}L_{2}F_{d}Q_{d}-d_{3}L_{U}-m_{3}L_{U}-d_{DC}L_{U};$$

Larvae feeding on infected host, $L_{I}$

(A5) $$\frac{dL_{I}}{dt}=m_{2}L_{2}F_{f}Q_{f}-d_{3}dL_{I}-m_{3}dL_{I}-d_{DC}dL_{I},$$

Uninfected engorged maturing larvae/young nymphs, $NU_{1}$

(A6) $$\frac{dNU_{1}}{dt}=m_{3}L_{U}+\left(1-p_{L}\right)\left(m_{3}L_{I}\right)-d_{L}NU_{1}-m_{L}NU_{1}-d_{WT}NU_{1},$$

Infected engorged maturing larvae/young nymphs, $NI_{1}$

(A7) $$\frac{dNI_{1}}{dt}=p_{L}\left(m_{3}L_{I}\right)-d_{L}NI_{1}-m_{L}NI_{1}-d_{WT}NI_{1},$$

Questing uninfected nymphs, $NU_{2}$

(A8) $$\frac{dNU_{2}}{dt}=m_{L}NU_{1}-d_{UN}NU_{2}-m_{n2}NU_{2}-d_{WT}NU_{2},$$

Questing infected nymphs, $NI_{2}$

(A9) $$\frac{dNI_{2}}{dt}=m_{L}NI_{1}-d_{UN}NI_{2}-m_{n2}NI_{2}-d_{WT}NI_{2},$$

Uninfected nymphs feeding on uninfected hosts, $FNU_{U}$

(A10) $$\frac{dFNU_{U}}{dt}=m_{n2}NU_{2}G_{d}Q_{d}-d_{fn}FNU_{U}-m_{fn}FNU_{U}-d_{DC}FNU_{U},$$

Uninfected nymphs feeding on infected hosts, $FNU_{I}$

(A11) $$\frac{dFNU_{I}}{dt}=m_{n2}NU_{2}G_{f}Q_{f}-d_{fn}FNU_{I}-m_{fn}FNU_{I}-d_{DC}FNU_{I},$$

Infected nymphs feeding on uninfected hosts, $FNI_{U}$

(A12) $$\frac{dFNI_{U}}{dt}=m_{n2}NI_{2}G_{d}Q_{d}-d_{fn}FNI_{U}-m_{fn}FNI_{U}-d_{DC}FNI_{U},$$

Infected nymphs feeding on infected hosts, $FNI_{I}$

(A13) $$\frac{dFNI_{I}}{dt}=m_{n2}NI_{2}G_{f}Q_{f}-d_{fn}FNI_{I}-m_{fn}FNI_{I}-d_{DC}FNI_{I},$$

Uninfected engorged maturing nymphs/young adults, $AU_{1}$

(A14) $$\frac{dAU_{1}}{dt}=m_{fn}\left(FNU_{U}\right)+m_{fn}\left(1-p_{N}\right)*\left(FNU_{I}\right)-f_{ND}AU_{1}-m_{N}AU_{1}-d_{WT}AU_{1},$$

Infected engorged maturing nymphs/young adults, $AI_{1}$

(A15) $$\frac{dAU_{1}}{dt}=m_{fn}\left(FNI_{I}\right)+m_{fn}FNI_{I}+m_{fn}\left(p_{N}\right)\left(FNU_{I}\right)-f_{ND}AI_{1}-m_{N}AI_{1}-d_{WT}AI_{1},$$

Questing uninfected adult, $AU_{2}$

(A16) $$\frac{dAU_{2}}{dt}=m_{N}AU_{1}-d_{UA}AU_{2}-m_{A2}AU_{2}-d_{WT}AU_{2},$$

Questing infected adult, $AI_{2}$

(A17) $$\frac{dAI_{2}}{dt}=m_{N}AI_{1}-d_{UA}AI_{2}-m_{A2}AI_{2}-d_{WT}AI_{2},$$

Uninfected adults feeding on uninfected hosts, $FAU_{U}$

(A18) $$\frac{dFAU_{U}}{dt}=m_{A2}AU_{2}H_{d}*Q_{d}-d_{A3}FAU_{U}-m_{A3}FAU_{U}-d_{DC}FAU_{U},$$

Uninfected adults feeding on infected hosts, $FAU_{I}$

(A19) $$\frac{dFAU_{I}}{dt}=m_{A2}AU_{2}H_{f}Q_{f}-d_{A3}FAU_{I}-m_{A3}FAU_{I}-d_{DC}FAU_{I},$$

Infected adults feeding on uninfected hosts, $FAI_{U}$

(A20) $$\frac{dFAI_{U}}{dt}=m_{A2}AI_{2}H_{d}Q_{d}-d_{A3}FAI_{U}-m_{A3}FAI_{U}-d_{DC}FAI_{U},$$

Infected adults feeding on infected hosts, $FAI_{I}$

(A21) $$\frac{dFAI_{I}}{dt}=m_{A2}AI_{2}H_{f}Q_{f}-d_{A3}FAI_{I}-m_{A3}FAI_{I}-d_{DC}FAI_{I},$$

Engorged adults, $A_{4}$

(A22) $$\frac{dA_{4}}{dt}=m_{A3}*\left(FAU_{U}+FAU_{I}+FAI_{U}+FAI_{I}\right)-m_{N}A_{4}-d_{WT}A_{4},$$

Gestating adults, $A_{5}$

(A23) $$\frac{dA_{5}}{dt}==m_{N}A_{4}-d_{A5}*A_{5}-d_{WT}A_{5},$$

Uninfected hosts, *U*

(A24) $$\frac{dU}{dt}=b_{H}\left(U+I\right)\left(1-\left(U+I\right)/K_{H}\right)-d_{H}U-J_{H},$$

Infected hosts, *I*

(A25) $$\frac{dI}{dt}=J_{H}-d_{HI}I,$$

### Appendix A.2. Auxiliary Equations

All nymphs and adults feeding on uninfected hosts, $T_{U}$

(A26) $$T_{U}=FNU_{U}+FNI_{U}+FAU_{U}+FAI_{U},$$

All nymphs and adults feeding on infected hosts

(A27) $$T_{I}=FNU_{I}+FNI_{I}+FAU_{I}+FAI_{I}$$

Percent available space per uninfected host weighted by probability ($q_{L}$) of larvae finding any host, $F_{d}$

(A28) $$F_{d}=max\left(q_{L}\left(CU-T_{U}\right)/\left(CU+\epsilon \right),0\right),$$

Percent available space per infected host weighted by probability ($q_{L}$) of larvae finding any host, $F_{f}$

(A29) $$F_{f}=max\left(q_{L}\left(CI-T_{I}\right)/\left(CI+\epsilon \right),0\right),$$

Percent available space per uninfected host weighted by probability ($q_{N}$) of nymph finding any host, $G_{d}$

(A30) $$Gd=max(q_{N}\left(CU-T_{U}\right)/\left(CU+\epsilon \right),0),$$

Percent available space per infected host weighted by probability ($q_{N}$) of nymph finding any host, $G_{f}$

(A31) $$Gf=max(q_{N}\left(CI-T_{I}\right)/\left(CI+\epsilon \right),0),$$

Percent available space per uninfected host weighted by probability ($q_{A}$) of adult finding any host, $H_{d}$

(A32) $$Hd=max(q_{A}\left(CU-T_{U}\right)/\left(CU+\epsilon \right),0),$$

Percent available space per infected host weighted by probability ($q_{A}$) of adult finding any host, $H_{f}$

(A33) $$Hf=max(q_{A}\left(CI-T_{I}\right)/\left(CI+\epsilon \right),0),$$

Total number of hosts of all types, *S*

(A34) S=U+I,

Fraction of hosts that are uninfected, $Q_{d}$

(A35) Qd=CUS/(S+P3d),

Fraction of hosts that are infected, $Q_{f}$

(A36) Qf=CIS/(S+P3f),

Transmission term for host infection, *J*

(A37) $$J=p_{UI}\left(FNI_{U}+FAI_{U}\right)U,$$

Temperature approximation for study area, *T*

(A38) $$\begin{array}{c} T=24.84+-8.501*cos(t*0.01721)+-1.668*sin(t*0.01721)+\\ -0.08626*cos(2*t*0.01721)+1.192*sin(2*t*0.01677), \end{array}$$

Percent humidity approximation for study area, *H*

(A39) $$\begin{array}{c} H=62.93+9.866*cos(t*0.01721)+-10.86*sin(t*0.01721)+\\ 3.166*cos(2*t*0.01721)+0.6116*sin(2*t*0.01721), \end{array}$$

### Appendix A.3. Parameters

**Table A1:** Model parameters.

Parameter	Description	Value	Units
*b*	oviposition rate	62.56	eggs per tick per day
$d_{e}$	daily death rate of eggs	0.015	percent per day
$d_{3}$	daily death rate of feeding larvae	=0.2	percent per day
$d_{fn}$	daily death rate of feeding nymphs	=0.01	percent per day
$d_{A3}$	daily death rate of feeding adults	0.01	percent per day
$d_{A5}$	daily death rate of gestating adults	0.0351	percent per day
$m_{1}$	young larvae to questing larvae maturation rate	0.069	percent per day
$m_{2}$	questing larvae to feeding larvae maturation	0.1	percent per day
$m_{3}$	larvae feeding on host maturation rate	0.232	percent per day
$m_{n2}$	uesting nymph maturation rate	0.1	percent per day
$m_{fn}$	feeding nymph maturation rate	0.142	percent per day
$m_{A2}$	questing adult maturation rate	0.1	percent per day
$m_{A3}$	feeding adult maturation rate	0.1058	percent per day
*C*	per host carrying capacity	50	maximum feeding nymphs and adults per host
$b_{H}$	birth rate of host	0.0135	per dog per day
$K_{H}$	carrying capacity of hosts	2000	number of dogs
$d_{H}$	death rate of uninfected host	0.0002739726	percent per day
$d_{HI}$	death rate of infected hosts	0.005479	percent per day
$p_{UI}$	probability of host infection by one feeding tick	0.0001	per infective tick per day
$p_{L}$	percent of feeding larvae infected	0.1	percent per day
$p_{N}$	percent of feeding nymphs infected	0.1	percent per day
ϵ	numerical feature	0.01	no units

## Appendix B. *Rhipicephalus sanguineus* Ticks Collected from Dogs during Home Inspections by Treatment (Pre and Post Intervention) for the Study Period, 2018–2019

**Treatment**	**# Time at** **Treatment**	**# Dogs** **Inspected**	**# Inspected** **Infested with** **Ticks**	***R. sanguineus*** **Larvae**	***R. sanguineus*** **Nymphs**	***R. sanguineus*** **Adults**	***Total Ticks*** Found per **Inspection Date**
**Pre Intervention**							
Control	Week 0	35	15	55	76	269	400
WIP1m	Week 0	20	14	22	17	885	924
WIP	Week 0	34	15	5	9	199	213
IRS-PPX	Week 0	28	4	0	0	17	17
**Post-Intervention**							
Control	Week 4	20	18	19	18	263	300
Control	Month 1	9	8	4	2	27	33
Control	Month 2	9	8	0	3	85	88
Control	Month 3	2	2	1	1	47	49
Control	Month 4	21	19	2	115	460	577
Control	Month 6	5	4	0	0	34	34
Control	Month 9	6	4	0	12	14	26
Control	Month 12	5	3	0	3	36	39
**Total**		77	66	26	154	966	
WIP1m	Week 4	16	6	0	0	11	11
WIP1m	Month 1	19	17	226	200	685	1111
WIP1m	Month 2	7	7	35	42	103	180
WIP1m	Month 3	10	10	181	498	150	829
WIP1m	Month 4	6	6	16	77	237	330
WIP1m	Month 6	9	6	9	33	208	250
WIP1m	Month 9	10	4	4	0	14	18
WIP1m	Month 12	9	7	24	107	154	285
**Total**		86	63	495	957	1562	
WIP1m	Week 4	12	6 1	1	42	44
WIP	Month 1	9	8	15	4	92	111
WIP	Month 2	10	7	0	11	79	90
WIP	Month 3	12	9	20	20	69	109
WIP	Month 4	12	7	0	4	27	31
WIP	Month 6	11	7	0	39	140	179
WIP	Month 9	16	13	3	40	31	74
WIP	Month 12	23	7	0	65	28	93
**Total**		105	64	39	184	508	
IRS-PPX	Week 4	13	1	0	0	2	2
IRS-PPX	Month 1	14	2	0	0	2	2
IRS-PPX	Month 2	13	10	0	1	67	68
IRS-PPX	Month 3	14	8	8	16	78	102
IRS-PPX	Month 4	7	7	8	1	28	37
IRS-PPX	Month 6	15	10	39	38	168	245
IRS-PPX	Month 9	21	8	0	1	25	26
IRS-PPX	Month 12	13	4	1	11	5	17
**Total**		110	50	56	68	375	
